# HIF-1α-dependent miR-424 induction confers cisplatin resistance on bladder cancer cells through down-regulation of pro-apoptotic UNC5B and SIRT4

**DOI:** 10.1186/s13046-020-01613-y

**Published:** 2020-06-10

**Authors:** Meng Yu, Toshinori Ozaki, Dan Sun, Haotian Xing, Baojun Wei, Jun An, Jieping Yang, Ying Gao, Shuangjie Liu, Chuize Kong, Yuyan Zhu

**Affiliations:** 1 The First Hospital of China Medical University, Shenyang, 110122 China; 2 Department of Reproductive Biology and Transgenic Animal, China Medical University, Shenyang, 110122 China; 3 Department of DNA Damage Signaling, Research Center, The 5th Hospital of Xiamen, Xiamen, 361101 Fujian China; 4 Department of Urology, The First Hospital of China Medical University, Shenyang, 110001 China; 5 Department of Urology, The General Hospital of Shenyang Military, Shenyang, 110016 China

**Keywords:** miR-424, UNC5B, SIRT4, HIF-1α, Cisplatin, Bladder cancer

## Abstract

**Background:**

Chemo-resistance of bladder cancer has been considered to be one of the serious issues to be solved. In this study, we revealed pivotal role of miR-424 in the regulation of CDDP sensitivity of bladder cancer cells.

**Methods:**

The cytotoxicity of cisplatin and effect of miR-424 were assessed by flow cytometry and TUNEL. Transcriptional regulation of miR-424 by HIF-1α was assessed by Chromatin immunoprecipitation (ChIP). Effect of miR-424 on expression of *UNC5B*, *SIRT4* (Sirtuin4) and apoptotic markers was measured by QRT-PCR and/or Western blot. The regulation of miR-424 for *UNC5B* and *SIRT4* were tested by luciferase reporter assay. The 5637-inoculated nude mice xenograft model was used for the in vivo study. The clinical significance of miR-424 was demonstrated mainly through data mining and statistical analysis of TCGA.

**Results:**

In this study, we have found for the first time that cisplatin (CDDP) induces the expression of miR-424 in a HIF-1α-dependent manner under normoxia, and miR-424 plays a vital role in the regulation of CDDP resistance of bladder cancer cells in vitro. Mechanistically, we have found that *UNC5B* and *SIRT4* are the direct downstream target genes of miR-424. CDDP-mediated suppression of xenograft bladder tumor growth was prohibited by the addition of miR-424, whereas ectopic expression of *UNC5B* or *SIRT4* partially restored miR-424-dependent decrease in CDDP sensitivity of bladder cancer 5637 and T24 cells. Moreover, knockdown of *UNC5B* or *SIRT4* prohibited CDDP-mediated proteolytic cleavage of PARP and also decreased CDDP sensitivity of these cells. Consistently, the higher expression levels of miR-424 were closely associated with the poor clinical outcome of the bladder cancer patients. There existed a clear inverse relationship between the expression levels of miR-424 and pro-apoptotic *UNC5B* or *SIRT4* in bladder cancer tissues.

**Conclusions:**

Collectively, our current results strongly suggest that miR-424 tightly participates in the acquisition/maintenance of CDDP-resistant phenotype of bladder cancer cells through down-regulation of its targets *UNC5B* and *SIRT4*, and thus combination chemotherapy of CDDP plus HIF-1α/miR-424 inhibition might have a significant impact on hypoxic as well as normoxic bladder cancer cells.

## Highlights

1. CDDP induces miR-424 expression in a HIF-1α-dependent manner in bladder cancer cells.
2. miR-424 confers CDDP resistance on bladder cancer cells in vitro and in vivo.
3. Pro-apoptotic *UNC5B* and *SIRT4* have been identified as new targets of miR-424.
4. miR-424 mediates CDDP resistance by inhibiting the expressions of *UNC5B* and *SIRT4*.
5. High expression levels of miR-424 predict a poor outcome in bladder cancer.

## Background

Bladder cancer is the fourth most common type of human urogenital cancer with the greatest mortality, which might be due to its high recurrence rates and wide-spread resistance to anti-cancer drugs [1]. Around 50% of patients benefit from pre- or post-surgery chemotherapy; however, recurrent disease often exhibits a serious chemo-resistant phenotype. It has been indicated that both of the intrinsic and the acquired resistance to chemotherapy might cause the extremely poor clinical efficacy of anti-cancer drugs against bladder cancer [2, 3]. To overcome these serious issues, it is urgent to understand the precise molecular mechanisms how bladder cancer cells could acquire and/or maintain the chemo-resistant phenotype, which might contribute to the development of a novel therapeutic strategy against refractory bladder cancer [4].

Accumulating evidence strongly suggests that the hypoxic conditions within bladder cancer tissues are tightly linked to their progression and chemo-resistant properties. For example, hypoxia-induced resistance to cisplatin (CDDP), doxorubicin (DOX), etoposide (VP16), melphalan (L-PAM), 5-flouoruracil (5-FU), gemcitabine (GEM) and docetaxel (DTX) has been described [5–8]. Under hypoxia, one of the early molecular events is hypoxia-mediated stabilization and activation of hypoxia-inducible factor-1α (HIF-1α), which triggers the development of the aggressive and the chemo-resistant phenotypes of numerous tumors [5]. HIF-1α transcription complex binds to the consensus hypoxia-responsive elements (HREs) “GCGTG” within its target gene promoters and transactivates its downstream target genes [5–8]. Alternatively, Cao et al. found that, under normoxia, certain anti-cancer drugs such as CDDP participate in the acquisition of the chemo-resistance as well as cancer stem cell enrichment [9–12]. Therefore, it is likely that there could exist a promising HIF-1α-target gene(s) implicated in the regulation of chemo-sensitivity.

MicroRNAs (miRNAs) are important small non-coding RNAs that regulate their target gene expression levels by inhibiting their mRNA translation into proteins. Of note, the deregulated expressions of certain miRNAs have been frequently detectable in a variety of human cancers including bladder cancer [13]. A growing body of evidence indicates that miRNAs might act as oncogenes or tumor-suppressor genes, which depends on their target genes. In general, oncogenic miRNAs that down-regulate their target tumor-suppressor gene expression, are aberrantly overexpressed in cancers, whereas miRNAs with tumor-suppressive potential that down-regulate their target oncogene expression, are significantly repressed in cancers [13]. Recently, it has been described that certain miRNAs are directly regulated by HIF-1α and participate in HIF-1α-mediated survival signaling pathway [6, 13, 14]. However, it remains elusive whether HIF-1α-target miRNAs could affect the chemo-sensitivity of cancer cells. Previously, Zhang et al. found that miR-424, a hypoxia-induced HIF-1α direct target which has an ability to stabilize HIF-1α, decreases the sensitivity of cancer cells to anti-cancer drugs such as DOX and VP16 through the inhibition of apoptotic cell death [15]. In addition, Berghmans et al. demonstrated that four miRNA signatures including miR-424 could predict the response rate of the advanced non-small cell lung carcinoma patients to CDDP-vinorelbine [16].

In the present study, we have described for the first time that HIF-1α-mediated induction of miR-424 causes down-regulation of pro-apoptotic* UNC5B* and *SIRT4,* thereby inhibiting CDDP-dependent apoptotic cell death of bladder cancer cells. Our current observations strongly suggest that miR-424 plays a pivotal role in the regulation of CDDP sensitivity of bladder cancer cells, and provides a clue to estimate the clinical efficacy of CDDP on bladder cancer patients.

## Materials and methods

### Cell culture

Human bladder carcinoma 5637, J82, BIU87 and T24 cells were purchased from the Chinese Academy of Sciences Committee on Culture Collection Cell Bank, Shanghai Institutes for Biological Sciences (Shanghai, China). Cells were cultured in RPMI 1640 medium (Invitrogen, Carlsbad, CA, USA) supplemented with 10% heat-inactivated fetal bovine serum (FBS, Invitrogen) and 1% glutamine at 37 °C in 5% CO_2_.

### Western blot analysis

Cells were lysed in lysis buffer (150 mM NaCl, 50 mM Tris-HCl, pH 7.5 and 0.5% NP-40) supplemented with the protease inhibitor cocktail (Sigma, Houston, TX, USA). Equal amounts of protein were separated by SDS-PAGE and transferred onto PVDF membranes (Millipore, Billerica, MA, USA). The membranes were probed with the primary antibody against UNC5B (abcam, Cambridge, UK), SIRT4 (Santa Cruz Biotechnology, Santa Cruz, CA, USA), PARP (Cell Signaling Technology, Beverly, MA, USA), cleaved caspase-3 (Millpore) or with β-actin (Sigma) for 1 h at room temperature. The membranes were then incubated with horseradish peroxidase (HRP)-conjugated goat anti-mouse or anti-rabbit IgG (Cell Signaling Technology) for 1 h at room temperature. Immuno-reactive signals were detected using an enhanced chemiluminescence system (ECL, Amersham Biosciences, Piscataway, NJ, USA).

### RNA extraction and quantitative real-time RT-PCR

Cells were washed in ice-cold PBS and mixed with 1 ml of TRIzol reagent (Invitrogen). Total RNA was extracted using the standard acid guanidinium thiocyanate–phenol–chloroform method. One microgram of total RNA was reverse-transcribed using MMLV reverse transcriptase according to the manufacturer’s instructions. Real-time quantitative reactions were carried out using gene-specific primers. The relative mRNA expression was calculated after normalizing with β-*actin* expression for each sample. For the quantitation of miRNAs, TaqMan MicroRNA reverse transcription kit was utilized according to the manufacturer’s instructions (Applied Biosystems, Foster City, CA, USA), and U6 was used as a control. Primers used are shown in Supplementary Table 1.

### Luciferase reporter assay

The wild-type (WT) 3′-untranslated region (3′-UTR) fragments containing the putative miR-424-binding sites were amplified from human *UNC5B* and *SIRT4*. The mutant 3′-UTR fragment (Mut) was amplified using the WT fragment as a template. These fragments were inserted into the downstream of Renilla luciferase reporter gene of psiCHECK-2 vector (Promega, Madison, WI, USA) to give psiCHECK2-*UNC5B*-WT, psiCHECK2-*UNC5B*-Mut, psiCHECK2-*SIRT4*-WT and psiCHECK2-*SIRT4*-Mut. Two hundred ninety three T cells were co-transfected with the indicated combinations of the luciferase reporter vectors and the expression vectors using Lipofectamine LTX (Invitrogen). Forty-eight hours after transfection, cell lysates were prepared and their luciferase activities (Firefly and renilla) were measured using the dual-luciferase reporter system according to the manufacturer’s instructions (Promega).

### Plasmid construction and lentiviral infection

To construct miR-424 expression vector, two oligonucleotides [5′-TCGACAGCAGCAATTCATGTTTTGAAGTGTGCTGTCCTTCAAAACATGAATTGCTGCTGTTTTT-3′ (forward) and 5′-AATTAAAAACAGCAGCAATTCATGTTTTGAAGGACAGCACACTTCAAAACATGAATTGCTGCTG-3′ (reverse)] were synthesized and cloned into pCDH-CMV lentiviral vector (System Biosciences, Mountain View, CA, USA). Lentiviral expression vectors for *UNC5B* and *SIRT4* were purchased from System Biosciences. 293 T cells were co-transfected with pPackH1 packaging plasmid mix (System Biosciences) and the indicated lentiviral vectors using Fugene HD transfection reagent following the manufacturer’s protocols (Promega). Forty-eight hours after transfection, the lentivirus particles were harvested.

### siRNA-mediated knockdown

siRNA against *UNC5B* or *SIRT4* was purchased from GenePharma (Shanghai, China). siRNAs were transfected into the indicated cells using Lipofectamine 2000 following the manufacturer’s instructions (Invitrogen).

### ChIP assay

T24 cells were cross-linked in 3.7% formaldehyde for 15 min, quenched in 0.125 M glycine for 5 min, and lysed with SDS lysis buffer. Chromatin was sheared by brief sonication, and lysates were precleared with salmon sperm DNA/protein A agarose beads (Millipore) for 1 h followed by the incubation with the control IgG or with the antibody against HIF-1α (Santa Cruz Biotechnology) in the presence of protein A agarose beads overnight. After the sequential wash, DNA was eluted in the elution buffer containing 1% SDS and 0.1 M NaHCO_3_, and cross-links were reversed by the addition of 0.2 M NaCl. DNA was purified by the standard phenol-chloroform extraction plus ethanol precipitation, and then analyzed by qPCR. Primer sequences were as follows: miR-424-F: 5′-GCAGCGGGCCAAGGCTGCGG-3′; miR-424-R: 5′-AACGCTCCCTTGGAGGCGAG-3′.

### In vivo xenograft model

Animal handling and experimental procedures were in accordance with the Guide for the Care and Use of Laboratory Animals, and approved by the Animal Experimental Ethics Committee of China Medical University. Five thousand six hundred thirty-seven cells (2 × 10^6^) were transduced with lentiviral vector encoding miR-424 (experimental group) or with control vector (control group), and inoculated subcutaneously into immunodeficient nude mice aged 3–4 weeks. Five mice were used in each group. The tumor volumes were measured twice a week and calculated based on the following formula: tumor volume (mm^3^) = ¼ length × width × height × 0.52.

Fourteen days after inoculation (around 200 mm^3^ of tumor volumes), mice of CDDP group were received intraperitoneal injection of CDDP (10 mg/kg) (Sigma) per day, whereas the control mice were received vehicle alone. Twenty-two days after inoculation, mice were sacrificed. Tumors were surgically removed, photographed, weighed and used for further pathological and histopathological evaluation.

### Apoptosis assay

At the indicated time points after the treatment, floating and adherent cells were harvested and washed in ice-cold PBS. Cells were stained with 5 μl of annexin V in 1× binding buffer for 15 min at room temperature in dark. After staining, 120 μl of 1× binding buffer plus 5 μl of propidium iodide were added to the cell suspension, and then cells were analyzed by FACS Calibur flow cytometer (FACS Canto II, BD Biosciences).

### TUNEL and immunohistochemistry staining

The tumor specimens, which were fixed in formaldehyde solution and embedded in paraffin, were cut into 4-μm-thick sections and then mounted on glass slides. Three independent tumors prepared from each group were subjected to TUNEL (Roche) and Ki67 (Santa Cruz Biotechnology)、UNC5B (abcam), SIRT4 (Santa Cruz Biotechnology), PARP (Cell Signaling Technology) staining. Data were expressed as the ratio of TUNEL- or Ki67-positive nuclei on total number of nuclei. Qualitative analysis of positive cells was carried out using the ImageJ-win64 image processing package.

### Determination of miR-424, *UNC5B* and *SIRT4* expressions in human tissue samples

Human bladder cancer samples were obtained from Department of Urology at the First Affiliated Hospital of China Medical University. The present studies were approved by the Ethics Committee (Institutional Review Board). The ages of all patients (including both men and women) were between 55 and 75 years. The cancer patients did not receive any neoadjuvant chemoradiotherapies prior to the surgical removal of the tumors. Total RNA was extracted from the fresh tissues using miRNeasy FFPE kit (Qiagen Inc., Hilden, Germany). Expression level of miR-424 was determined by quantitative real-time PCR (qRT-PCR) as described above. Clinicopathological characters of the patients and tissues used were described in Supplementary Table 2.

### Analysis of 2011 TCGA data set

A normalized RNA-seq dataset of bladder cancers in the Cancer Genome Atlas (TCGA) open-access database was downloaded from the cBioPortal for cancer genomics and used to evaluate miR-424 transcript levels. This dataset includes miRNA profiles of 407 bladder cancer patients. The staging and grading evaluation followed the American Joint Committee on Cancer 8th TNM staging system and.

World Health Organization (WHO) 2004/2016 classification, respectively. Differences were considered significant at *P* < 0.05.

### Statistical analysis

The data were presented as the mean ± S.D. The samples were analyzed using a two-tailed unpaired Student’s *t*-test, Kaplan-Meier plot and Pearson correlation coefficient. *P*-values < 0.05 were considered statistically significant.

## Results

### CDDP induces miR-424 expression in a HIF-1α-dependent manner

**Fig. 1:**
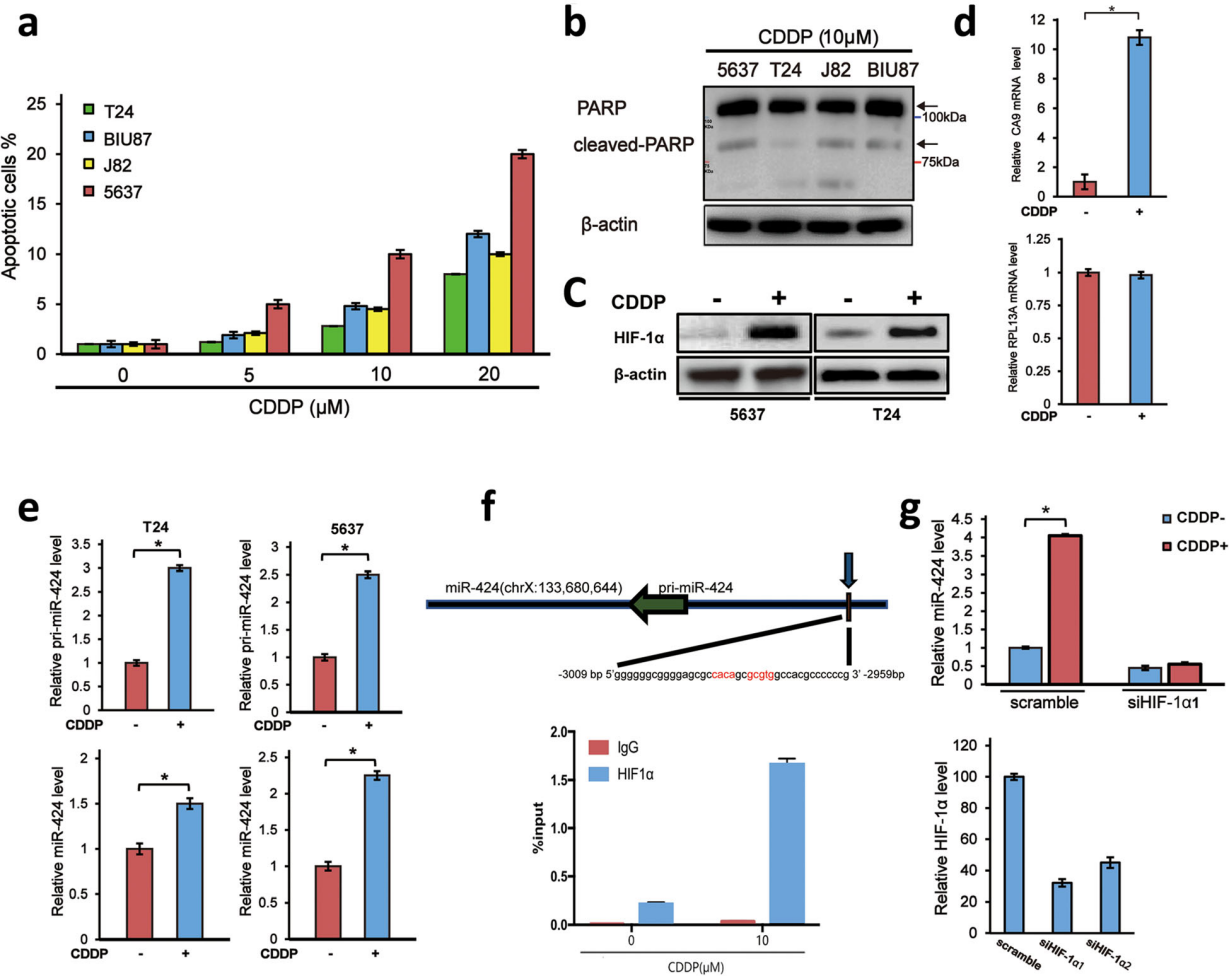
CDDP induces miR-424 in bladder cancer cells in a HIF-1α-dependent manner. **a** FACS analysis. Bladder cancer 5637, J82, BIU87 and T24 cells were treated with the increasing concentrations of CDDP. Twenty-four hours after treatment, cells were subjected to FACS analysis. **b** CDDP-mediated cleavage of PARP. The indicated cells were exposed to 10 μM of CDDP. Twenty-four hours after exposure, cell lysates were prepared and analyzed by Western blotting. β-actin was used as a loading control. **c** CDDP-mediated induction of HIF-1α. Five thousand six hundred thirty-seven (left panels) and T24 (right panels) cells were treated with 10 μM of CDDP or left untreated. Twenty-four hours after treatment, cell lysates were analyzed for HIF-1α by Western blotting. β-actin was used as a loading control. **d** CDDP specifically potentiates HIF-1α transcriptional activity. T24 cells were treated with 10 μM of CDDP or left untreated. Twenty-four hours after treatment, total RNA was prepared and analyzed for *CA9* and *RPL13A* by qRT-PCR. **e** CDDP-dependent up-regulation of miR-424. T24 (left panels) and 5637 (right panels) cells were treated as in (**c**). Twenty-four hours after treatment, total RNA was prepared and analyzed for pri-miR-424 (upper panels) and miR-424 (lower panels) by qRT-PCR. **f** Chromatin immunoprecipitation (ChIP). T24 cells were exposed to 10 μM of CDDP or left untreated. Cross-linked chromatin was immunoprecipitated with the control IgG or with anti-HIF-1α antibody, and the immunoprecipitated DNA was subjected to qPCR using primers that flanked the putative HIF-1α-binding site. The results were normalized to T24 cells without CDDP treatment, in which ChIP was carried out with the control IgG (mean ± SEM; n = 3). The nucleotide sequence of the HIF-1α-binding site is also shown. **g** HIF-1α-dependent induction of miR-424 in response to CDDP. Five thousand six hundred thirty-seven cells were transfected with the scramble siRNA or with siRNA against *HIF-1α* and treated with or without 10 μM of CDDP. Knockdown efficiency of HIF-1α siRNAs was validated by qRT-PCR (left panel). Twenty-four hours after CDDP treatment, total RNA was prepared and subjected to qRT-PCR. *P < 0.05, **P < 0.01

Although CDDP is one of the most effective chemotherapeutic agents for bladder cancer, its intrinsic and/or acquired drug resistance has been considered to be a major obstacle. To elucidate the molecular mechanisms behind CDDP resistance of bladder cancer, we asked the possible effect of CDDP on the indicated bladder cancer cells (T24, BIU87, J82 and 5637 cells). As shown in Fig. 1a, CDDP-induced cell death was detected in these four bladder cancer cells in a dose-dependent manner. Among them, 5637 and T24 cells displayed the highest and the lowest sensitivity to CDDP, respectively. Consistent with these observations, CDDP-mediated proteolytic cleavage of PARP in 5637 cells was stronger than that of T24 cells (Fig. 1b). Based on these results, we have employed 5637 and T24 cells for further experiments.

Since anti-cancer drugs such as CDDP, doxorubicin (DOX), paclitaxel (PTX), gemcitabine (GEM) and carboplatin (CBDCA), stimulate pro-survival HIF-1α and its downstream target gene expression under normoxia [9–12], it is likely that HIF-1α is responsible for the acquisition and/or the maintenance of the chemo-resistant phenotypes of bladder cancer cells. As shown in Fig. 1c, CDDP caused a significant increase in HIF-1α expression in 5637 and T24 cells. Under our experimental conditions, HIF-1α-target gene *CA9* but not HIF-1α-unrelated *RPL13A* was induced in T24 cells following CDDP exposure (Fig. 1d), indicating that CDDP enhances the expression and the transcriptional activity of HIF-1α in bladder cancer cells.

It has been shown that miR-424, a hypoxia-induced HIF-1α direct target miRNA, has an ability to stabilize HIF-1α and thus reduces the anti-cancer drug sensitivity through the inhibition of apoptotic cell death in lung cancer cells [15], raising a possibility that HIF-1α/miR-424 axis might participate in the regulation of CDDP sensitivity in bladder cancer. To test this possibility, we have examined the expression levels of miR-424 and its primary transcript (pri-miR-424) in T24 and 5637 cells. As shown in Fig. 1e, CDDP-exposed T24 and 5637 cells expressed a larger amount of pri-miR-424 and miR-424 relative to non-treated cells, indicating that CDDP positively regulates miR-424 transcription in these bladder cancer cells. In support of these observations, ChIP assay demonstrated that HIF-1α is efficiently recruited onto the putative HIF-1α-binding site within 5′-upstream region of miR-424 gene in the presence of CDDP (Fig. 1f). Furthermore, siRNA-mediated knockdown of *HIF-1α* strongly prohibited CDDP-dependent up-regulation of miR-424 in T24 cells (Fig. 1g).

Together, these results suggest that CDDP-mediated up-regulation of miR-424 is modulated in a HIF-1α-dependent manner.

### MiR-424 confers CDDP resistance on bladder cancer cells in vitro

**Fig. 2:**
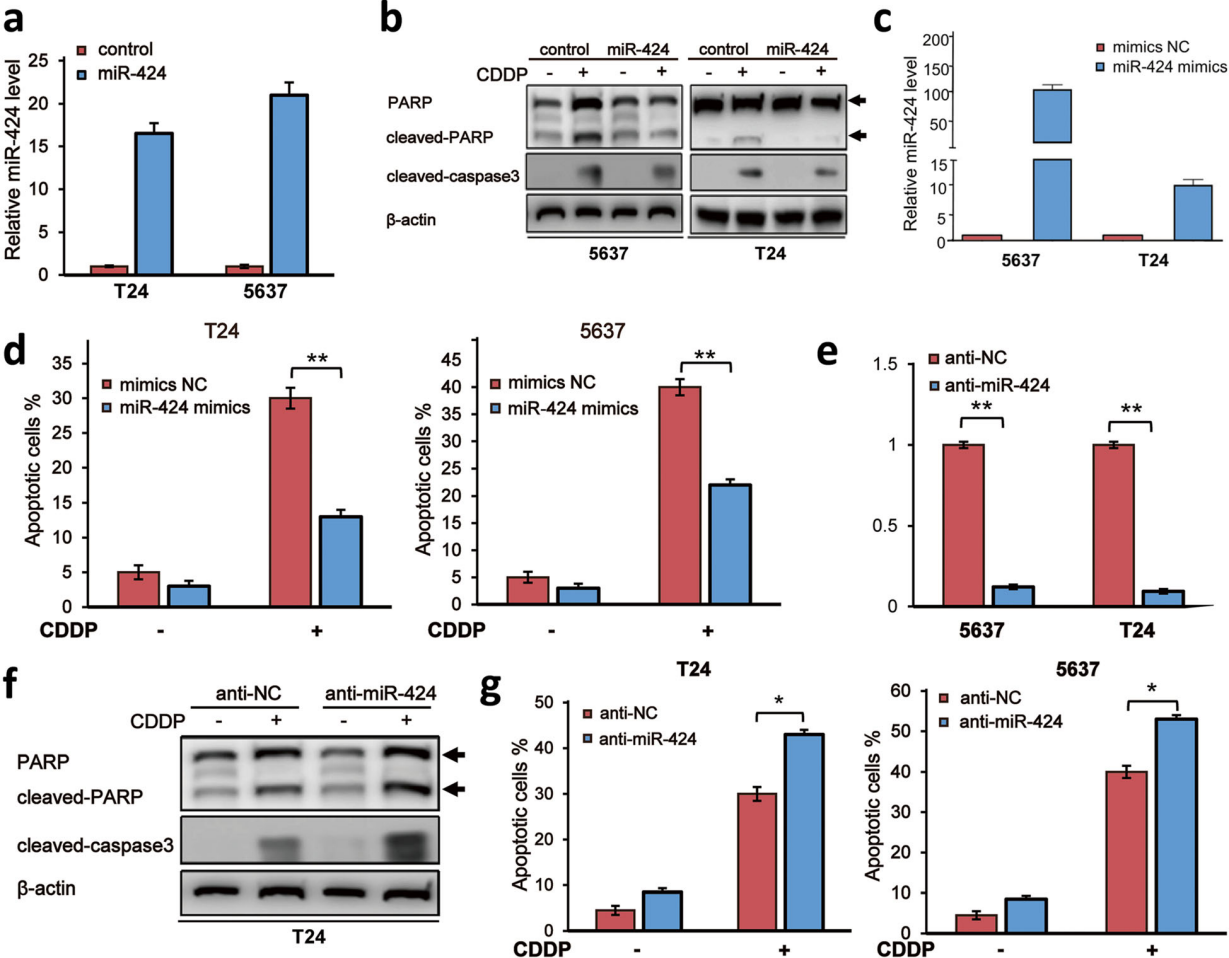
Forced expression of miR-424 attenuates CDDP-mediated apoptotic cell death of bladder cancer cells. **a** Forced expression of miR-424. T24 and 5637 cells were transfected with the empty vector or with the expression vector for miR-424. Forty-eight hours after transfection, the expression level of miR-424 was examined by qRT-PCR. **b** miR-424 prohibits CDDP-induced proteolytic cleavages of PARP and caspase-3. Five thousand six hundred thirty-seven (left panels) and T24 (right panels) cells were transfected and treated as in (**a**). Twenty-four hours after treatment, cell lysates were analyzed by Western blotting with the indicated antibodies. β-actin was used as a loading control. **c** Overexpression of miRNA-424 mimics. Five thousand six hundred thirty-seven and T24 cells were transfected with the negative control (NC) or with miR-424 mimics. Forty-eight hours after transfection, the expression level of miR-424 was examined by qRT-PCR. **d** miR-424 mimics prohibit CDDP-induced apoptotic cell death. T24 (left panel) and 5637 (right panel) cells were transfected with the scrambled oligo or with miRNA-424 mimics followed by CDDP exposure (10 μM). Twenty-four hours after treatment, cells were subjected to FACS analysis. **e** miR-424 inhibitor reduces the expression level of miR-424. Five thousand six hundred thirty-seven and T24 cells were transfected with the control or with miR-424 inhibitor. Twenty-four hours after transfection, the expression level of miR-424 was examined by qRT-PCR. **f** miR-424 inhibitor augments CDDP-induced proteolytic cleavages of PARP and caspase-3. T24 cells were transfected with the scrambled oligo or with miRNA-424 inhibitor, and then exposed to CDDP (10 μM). Twenty-four hours after treatment, cell lysates were prepared and analyzed by Western blotting with the indicated antibodies. β-actin was used as a loading control. **g** CDDP-mediated apoptotic cell death is further enhanced by miR-424 inhibitor treatment. T24 (left panel) and 5637 (right panel) cells were transfected and treated as in (**e**). Twenty-four hours after treatment, cells were processed for FACS analysis. The data are presented as the mean ± S.D. of triplicate experiments. *P < 0.05, **P < 0.01

To address the biological significance of miR-424 in bladder cancer cells exposed to CDDP, we have examined the possible effect of miR-424 on CDDP-induced apoptotic cell death of 5637 and T24 cells. As shown in Fig. 2a and b, ectopic expression of miR-424 attenuated CDDP-dependent proteolytic cleavages of caspase-3 and PARP, and forced expression of miR-424 mimics significantly decreased number of CDDP-induced apoptotic cells (Fig. 2c, d). In accordance with these results, miR-424 inhibitor (antisense oligomer) treatment strongly prohibited the expression of the endogenous miR-424 in 5637 and T24 cells (Fig. 2e), and enhanced CDDP-mediated cleavages of caspase-3 and PARP in T24 cells with a lower sensitivity to CDDP (Fig. 2f). As expected, FACS analysis demonstrated that number of cells with sub-G1 DNA content was markedly increased in miR-424 inhibitor-treated 5637 and T24 cells relative to that of untreated cells (Fig. 2g).

Together, these observations strongly suggest that miR-424 attenuates CDDP-dependent apoptotic cell death in bladder cancer cells.

### MiR-424 mediates CDDP resistance by regulating its downstream targets *UNC5B* and *SIRT4*

**Fig. 3:**
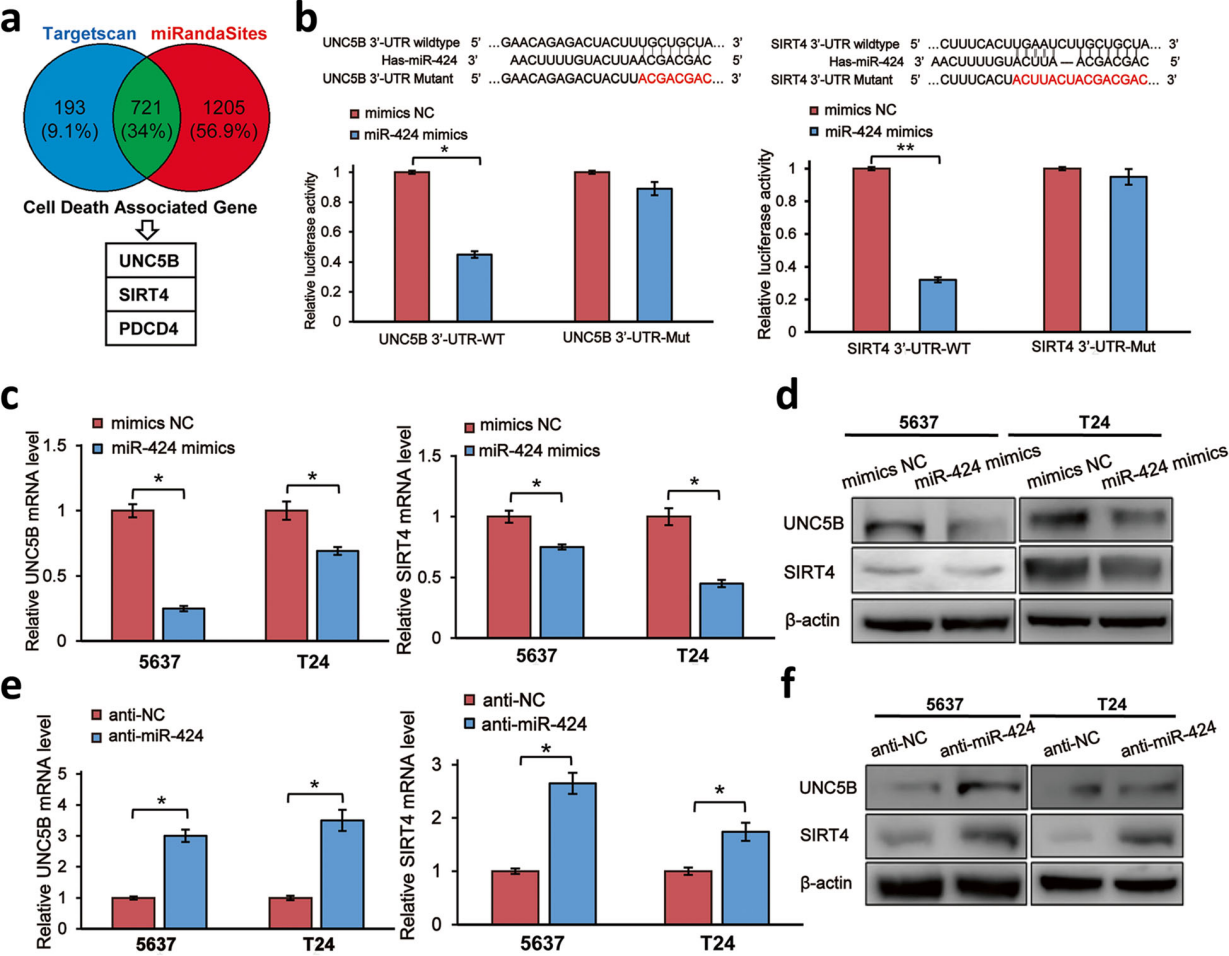
*UNC5B* and *SIRT4* are the direct downstream targets of miR-424. **a** Potential downstream targets of miR-424 implicated in apoptotic cell death. **b** Luciferase reporter assay. The putative miR-424-binding sequence within 3′-UTR of *UNC5B* or *SIRT4* are shown, and the mutated nucleotides are indicated in red (upper). For luciferase reporter assays, T24 cells were co-transfected with the indicated luciferase reporter constructs together with miR-424 mimics or with the scramble miRNA. Forty-eight hours after transfection, their luciferase activities were measured (lower). **c** miR-424 down-regulates *UNC5B* and *SIRT4*. Five thousand six hundred thirty-seven and T24 cells were transfected with miR-424 mimics or with the scramble miRNA. Forty-eight hours after transfection, total RNA was prepared and analyzed for *UNC5B* (left panel) and *SIRT4* (right panel) by qRT-PCR. **d** miR-424 down-regulates UNC5B and SIRT4 at protein level. Five thousand six hundred thirty-seven and T24 cells were transfected as in (**c**). Forty-eight hours after transfection, cell lysates were extracted and subjected to Western blotting with the indicated antibodies. β-actin was used as a loading control. **e** Inhibition of miR-424 promotes *UNC5B* and *SIRT4* transcription. Five thousand six hundred thirty-seven and T24 cells were transfected with the negative control or with miR-424 inhibitor. Forty-eight hours after transfection, total RNA was prepared and analyzed for *UNC5B* (left panel) and *SIRT4* (right panel) by qRT-PCR. **f** Inhibition of miR-424 increases UNC5B and SIRT4 at protein level. Five thousand six hundred thirty-seven and T24 cells were transfected as in (**e**). Forty-eight hours after transfection, cell lysates were extracted and subjected to Western blotting with the indicated antibodies. β-actin was used as a loading control. *P < 0.05, **P < 0.01

To elucidate the molecular mechanisms how miR-424 could participate in the acquisition and/or the maintenance of CDDP resistance in bladder cancer cells, we sought to identify its downstream effectors using the web-based software TargetScan and miRanda. From the extensive analysis, we have finally identified apoptosis-related *UNC5B* and *SIRT4* as the potential downstream targets of miR-424 (Fig. 3a). To check whether *UNC5B* and/or *SIRT4* could be regulated by miR-424, the dual-luciferase UTR vectors carrying the wild-type 3′-UTR of *UNC5B* (*UNC5B* 3′-UTR-WT), the wild-type 3′-UTR of *SIRT4* (*SIRT4* 3′-UTR-WT), the mutant 3′-UTR of *UNC5B* (*UNC5B* 3′-UTR-Mut) or the mutant 3′-UTR of *SIRT4* (*SIRT4* 3′-UTR-Mut), in which the putative miR-424-binding site was mutated, were constructed for luciferase reporter assays. T24 cells were transiently transfected with the indicated luciferase reporter vectors together with the negative control or with miR-424 mimics. As seen in Fig. 3b, miR-424 mimics obviously reduced the luciferase activity driven by *UNC5B* 3′-UTR-WT and *SIRT4* 3′-UTR-WT. By contrast, miR-424 mimics had an undetectable effect on the luciferase activity driven by *UNC5B* 3′-UTR-Mut and *SIRT4* 3′-UTR-Mut. Consistent with these observations, forced expression of miR-424 mimics significantly reduced the endogenous *UNC5B* and *SIRT4* expressions at mRNA and protein levels in 5637 and T24 cells (Fig. 3c, d). Meanwhile, miR-424 inhibitor treatment resulted in a remarkable increase in* UNC5B* and* SIRT4* at mRNA and protein levels (Fig. 3e, f).

**Fig. 4:**
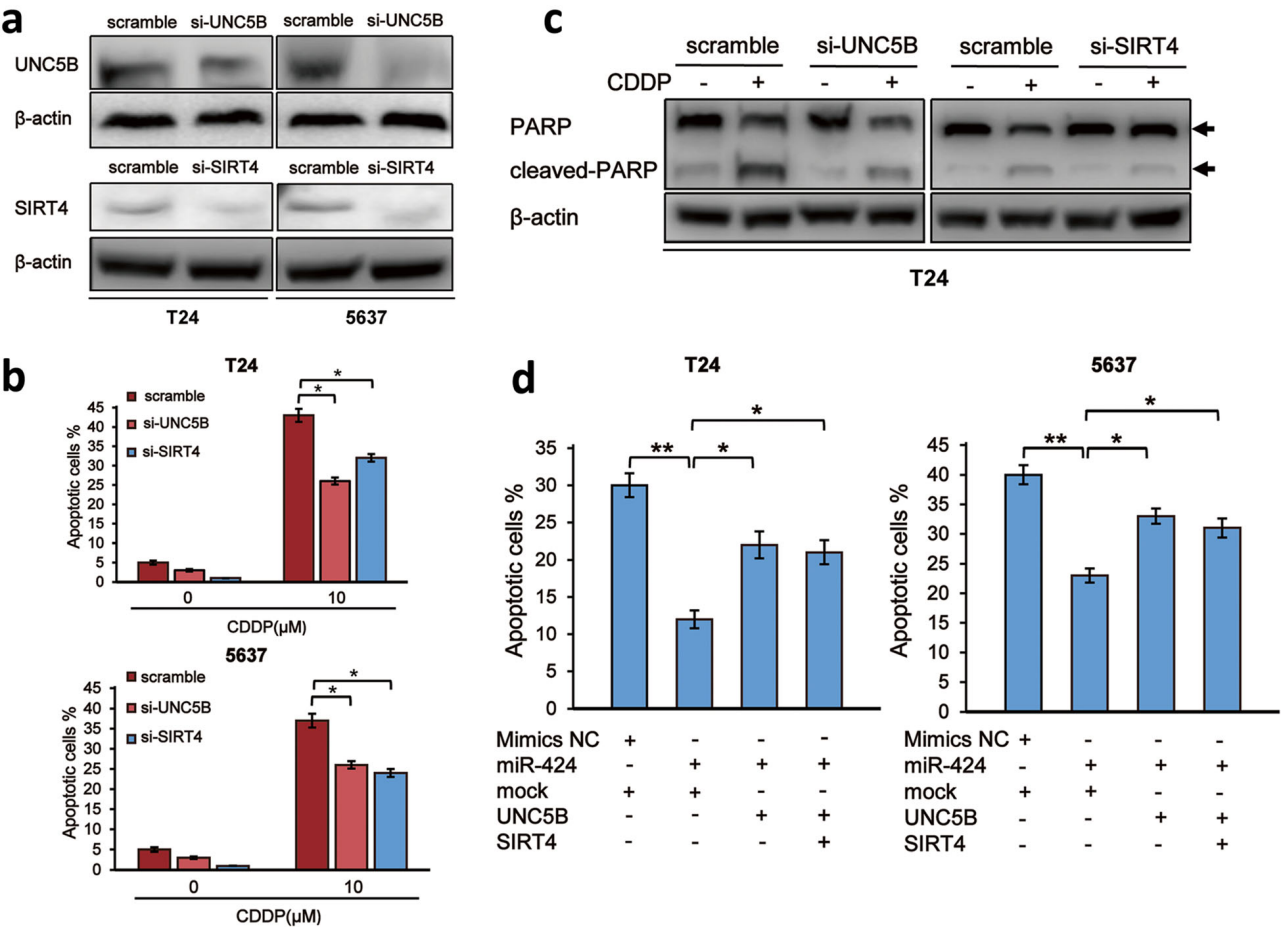
Depletion of *UNC5B* or *SIRT4* suppresses CDDP-mediated apoptotic cell death. **a** Knockdown of *UNC5B* or *SIRT4*. T24 (left panels) and 5637 (right panels) cells were transfected with the non-targeting scramble siRNA or with siRNA against *UNC5B* or *SIRT4*. Forty-eight hours after transfection, cell lysates were extracted and analyzed for UNC5B (upper panels) and SIRT4 (lower panels) by Western blotting. β-actin was used as a loading control. **b** Silencing of *UNC5B* or *SIRT4* decreases CDDP sensitivity of bladder cancer cells. T24 (left panel) and 5637 (right panel) cells were transfected as in (**a**). Twenty-four hours after transfection, cells were treated with 10 μM of CDDP or left untreated. Twenty-four hours after treatment, cells were subjected to FACS analysis. (**c**) Knockdown of *UNC5B* or *SIRT4* attenuates CDDP-induced proteolytic cleavage of PARP. T24 cells were transfected and treated as in (**b**). Twenty-four hours after CDDP treatment, cell lysates were prepared and subjected to Western blotting with anti-PARP antibody. β-actin was used as a loading control. **d** miR-424-caused suppression of CDDP-mediated apoptotic cell death is partially restored by ectopic expression of UNC5B or SIRT4. T24 (left panel) and 5637 (right panel) cells were transfected with the indicated combinations of the expression vectors. Twenty-four hours after transfection, cells were exposed to 10 μM of CDDP. Twenty-four hours after treatment, cells were processed for FACS analysis. The data are presented as the mean ± S.D. of triplicate experiments. *P < 0.05, **P < 0.01

It has been shown that *UNC5B* and *SIRT4* play a vital role in the regulation of numerous cellular processes such as cell survival, metabolism and apoptotic cell death [17–23]. To investigate the functional roles of miR-424-mediated down-regulation of *UNC5B* and/or *SIRT4* in CDDP-induced apoptotic cell death of bladder cancer cells, siRNA-mediated gene silencing of *UNC5B* and *SIRT4* was performed in T24 and 5637 cells (Fig. 4a). As seen in Fig. 4b, *UNC5B*- or *SIRT4*-knockdown significantly decreased number of apoptotic cells in response to CDDP. In accordance with these observations, Western blot analysis demonstrated that CDDP-dependent proteolytic cleavage of PARP is attenuated in *UNC5B*- or *SIRT4*-knocked down T24 cells (Fig. 4c). These results suggest that* UNC5B* and *SIRT4* are required at least in part for CDDP-induced apoptotic cell death of bladder cancer cells, and that miR-424 confers CDDP resistance on bladder cancer cells through down-regulation of *UNC5B* and *SIRT4*. In support of this notion, miR-424 mimics-mediated decrease in CDDP sensitivity of T24 and 5637 cells was partially restored by forced expression of *UNC5B* or *SIRT4* lacking miR-424-responsive 3′-UTR (Fig. 4d).

### miR-424 reduces the efficacy of CDDP on xenograft tumor growth

**Fig. 5:**
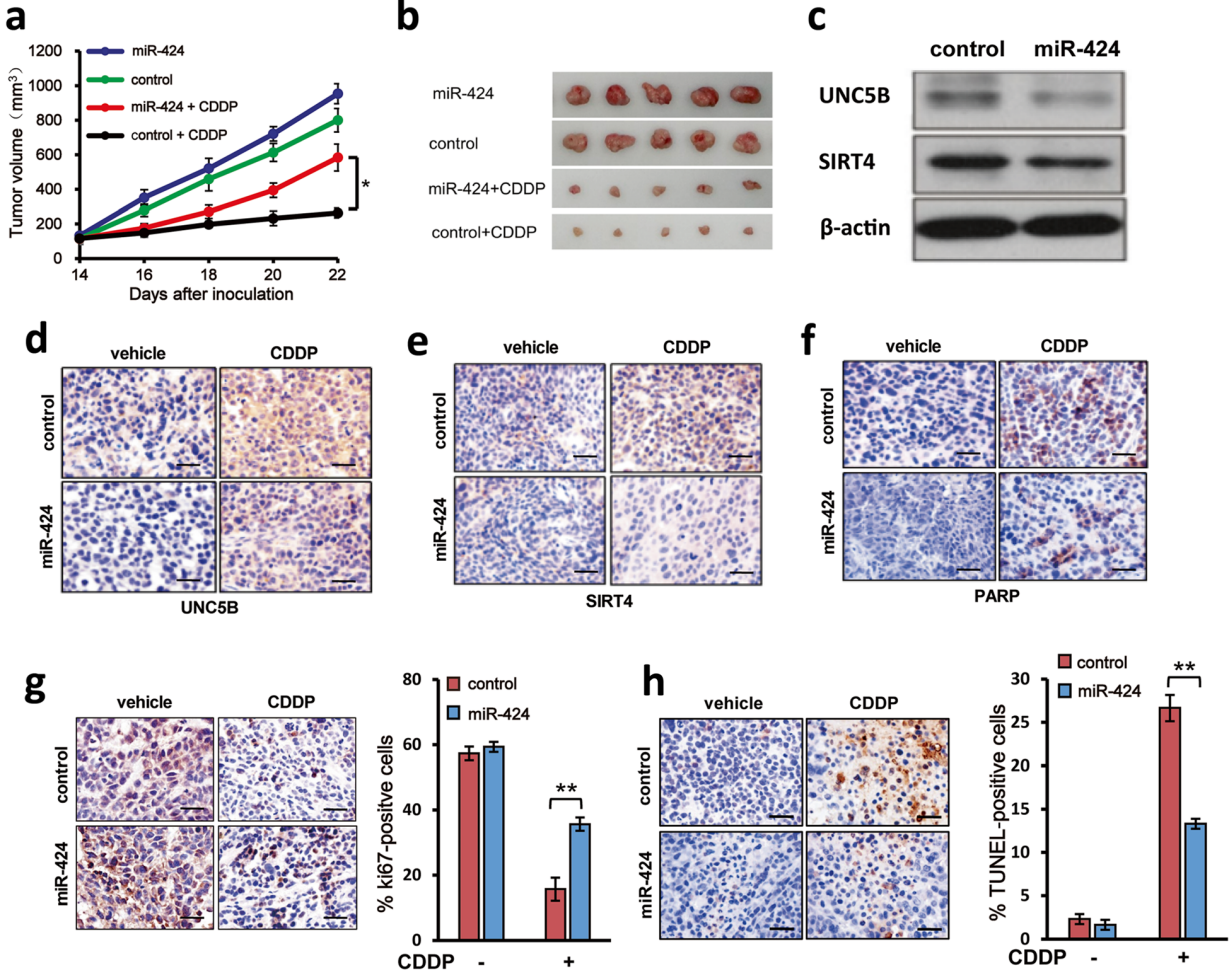
miR-424 decreases the sensitivity of bladder cancer cells to CDDP in vivo*.* Five thousand six hundred thirty-seven cells transduced with the empty lentiviral vector or with the lentiviral vector for miR-424 were inoculated into nude mice. Fourteen days after inoculation, mice were treated with CDDP or with vehicle alone. At the indicated time points after inoculation, volumes of the tumors were measured (**a**). The representative pictures of the surgically removed tumors of each group were taken (**b**). **c** Western blot. Cell lysates prepared from the indicated xenograft tumor tissues were subjected to Western blot with the indicated antibodies. β-actin was used as a loading control. **d-f** Immunohistochemical staining. Paraffin-embedded sections prepared from the indicated xenograft tumor tissues were analyzed for UNC5B (D), SIRT4 (**e**) and PARP (**f**) by immunohistochemical staining. (G-H) miR-424 expression increases and decreases number of Ki67-positive and TUNEL-positive cells, respectively. The formaldehyde-fixed paraffin-embedded xenograft tumors were cut into 4-μm-thick sections and stained with anti-Ki67 antibody (**g**) or with TUNEL (**h**). % Ki67- or TUNEL-positive cells were shown (right panels). The data are presented as the mean ± S.E. *P < 0.05, **P < 0.01. Scale bars, 25 μm

To further confirm the role of miR-424 in the regulation of CDDP sensitivity of bladder cancer cells, 5637 cells transduced with the control lentiviral vector or with the lentiviral vector for miR-424 were subcutaneously inoculated into athymic nude mice. Tumors were allowed to grow for 14 days prior to the initiation of the experiments. Mice were then treated with CDDP (10 mg/kg per day) or with the vehicle alone. At the indicated time points after CDDP treatment, tumor volumes of each group were measured. As shown in Fig. 5a and b, compared to the control group treated with the vehicle alone, CDDP treatment significantly attenuated tumor growth of the control as well as miR-424 overexpressing group. Of note, miR-424 overexpressing group had a larger tumor volume than that of the control group expressing the scramble miRNA (*P* < 0.01). Consistent with our in vitro results, the expression levels of *UNC5B* and *SIRT4* were reduced in CDDP-treated xenograft tumors overexpressing miR-424 (*P* < 0.01, respectively; Fig. 5c–e). In CDDP treatment group, 86.2 ± 12.2 and 74.5 ± 14.6 labeled cells detected for UNC5B and SIRT4 respectively. In CDDP-treated xenograft tumors overexpressing miR-424 group,42.4 ± 10.6 and 34.6 ± 11.5 cells were marked for UNC5B and SIRT4 respectively, compared with the control groups that were 24.4 ± 4.6 and 20.6 ± 6.2 labeled cells for UNC5B and SIRT4 respectively, while 18.1 ± 7.5 cells and 17.3 ± 8.2 cells were marked for UNC5B and SIRT4 respectively in miR-424 overexpressing group (Fig. 5d, e). Moreover, the amount of cleaved PARP was also decreased in CDDP-treated miR-424-overexpressing tumors (12.6 ± 4.1%)as compared to that in the tumors exposed to CDDP (28.2 ± 5.6%) (*P* < 0.05),while compared to the vehicle (3.6 ± 1.3%). miR-424 overexpressing (2.2 ± 0.8%) slightly effected the expression of cleaved PARP-1 (Fig. 5f). In accordance with these observations, immunohistochemical analyses revealed that number of Ki67-positive cells was increased in CDDP-exposed tumors overexpressing miR-424 relative to that in the control tumors (Fig. 5g). Additionally, forced expression of miR-424 led to a marked decrease in number of TUNEL-positive cells (Fig. 5h). Collectively, these results indicate that miR-424 suppresses CDDP-mediated bladder cancer cell death through down-regulation of UNC5B and SIRT4 in vivo.

### The expression level of miR-424 is inversely correlated with those of *UNC5B* and *SIRT4* in bladder cancer

**Fig. 6:**
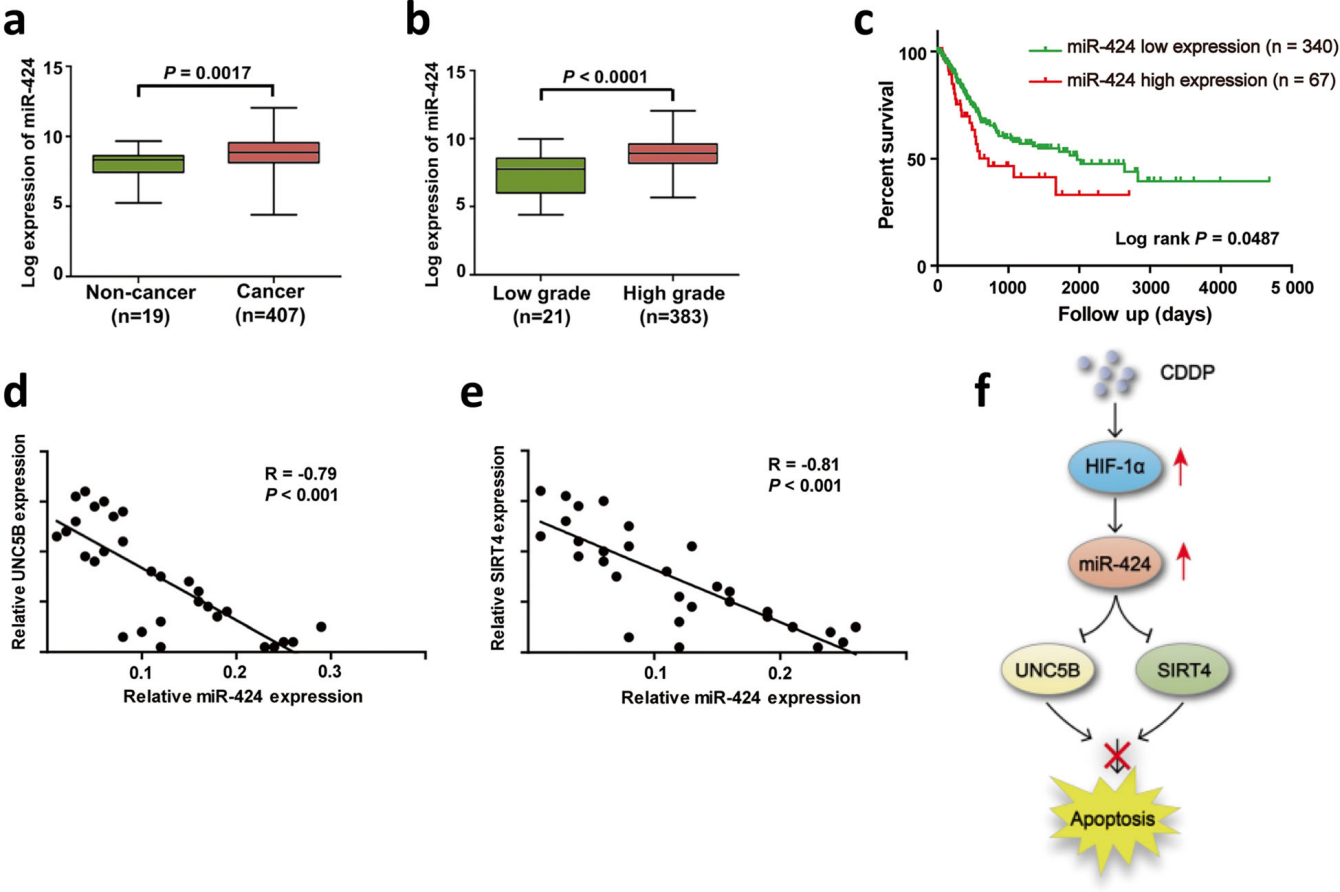
The expression level of miR-424 is inversely correlated with those of *UNC5B* and *SIRT4* in bladder cancer. **a** miR-424 expression in bladder cancer tissues vs. normal ones. An open-access TCGA RNA-seq dataset containing 407 bladder cancers and 19 normal controls was employed in the present analysis. **b** miR-424 expression in low-grade bladder cancers *vs.* high-grade bladder cancers. The dataset in (**a**) was used in this analysis. **c** Kaplan-Meier survival analysis of TCGA bladder cancer dataset as in (**a**). **d**, **e** The correlation between the expression levels of miR-424 and *UNC5B* (D) or between those of miR-424 and *SIRT4* (**e**) in an independent bladder cancer cohort (n = 30). **f** The working model of miR-424-mediated regulation of CDDP resistance of bladder cancer. Upon CDDP, HIF-1α is rapidly activated and up-regulates miR-424, which down-regulates pro-apoptotic *UNC5B* and *SIRT4*, and thereby bladder cancer cells become CDDP-resistant

Since it has been described that miR-424 expression level is increased in certain human primary tumors [24–26], we sought to examine the expression level of miR-424 in bladder cancer. To this end, we analyzed the expression level of miR-424 by taking advantage of the TCGA public RNA-seq database. As shown in Fig. 6a and b, miR-424 expression level was elevated in human bladder cancer tissues compared to their corresponding paracancerous normal ones (*P* = 0.0017), and significantly increased in high-grade (poorly differentiated) tumors compared to low-grade (differentiated) ones. We also found that the expression of miR-424 was increased in patients with high stage (T3 and T4), but the difference was not statistically significant (*P* = 0.0641). There was no significant difference in the expression of miR-424 between patients with non-lymph node metastasis and patients with lymph node metastasis (*P* = 0.1685). Furthermore, Kaplan-Meier plot demonstrated that a higher expression level of miR-424 is closely associated with the poor prognosis of the bladder cancer patients (*P* = 0.0487, Fig. 6c). Next, we asked whether there could exist the correlation between miR-424 and *UNC5B* or *SIRT4* expression levels in our bladder cancer samples (n = 30). As shown in Fig. 6d and e, there existed a clear inverse relationship between the expression levels of miR-424 and that of *UNC5B* or *SIRT4* (*Pearson correlationr *= − 0.79, *P* < 0.001 and *r *= − 0.81, *P* < 0.001, respectively). Taken together, these results strongly suggest that the enhanced expression of miR-424 plays a deleterious role in bladder cancer through down-regulating *UNC5B* and/or *SIRT4*.

## Discussion

In the current study, we have found for the first time that miR-424, a direct target of HIF-1α, decreases CDDP sensitivity of bladder cancer cells through down-regulation of pro-apoptotic *UNC5B* and *SIRT4* (Fig. 6f). Our present results unveiled the molecular mechanisms how bladder cancer cells acquire and/or maintain CDDP resistance under normoxia, and also might provide a clue to develop a promising strategy to treat CDDP-resistant bladder cancer patients.

miR-424 has been initially identified as a differentiation-specific miRNA, which played an important role in the regulation of the monocyte/macrophage differentiation program [27]. In addition to differentiation, a growing body of evidence suggests that miR-424 has a dual function (oncogene or tumor-suppressor) during carcinogenesis, dependent on its target genes [28–33]. According to our present results, miR-424 was significantly increased in bladder cancer tissues, especially in high-grade tumors, and its higher expression level was associated with the poor clinical outcome, indicative of its oncogenic role in bladder cancer. Forced expressions of miR-424 and its mimics conferred CDDP resistance on bladder cancer 5637 and T24 cells in vitro and in vivo, while miR-424 inhibitor enhanced the cytotoxic activity of CDDP in these bladder cancer cells, suggesting that miR-424 contributes to the acquired CDDP resistance of bladder cancer. Notably, CDDP treatment induced miR-424 expression in a HIF-1α-dependent manner in bladder cancer 5637 cells. Collectively, our findings imply that targeting miR-424 might be an effective therapeutic strategy against refractory bladder cancers.

Another new finding of the present study was that pro-apoptotic *UNC5B* and *SIRT4* are novel downstream target genes of miR-424. Based on our present results, forced expression of miR-424 mimics reduced *UNC5B* and *SIRT4* expressions in bladder cancer 5637 and T24 cells, whereas miR-424 inhibitor increased *UNC5B* and *SIRT4* expressions in these cells. Consistent with these results, we have identified a potential miR-424-binding site within the 3′-UTR of *UNC5B* and *SIRT4*. Luciferase reporter assay revealed that miR-424 remarkably prohibits the reporter activity driven by the wild-type but not the mutated 3′-UTR of these genes, suggesting that *UNC5B* and *SIRT4* are the direct downstream targets of miR-424. Inverse correlations between miR-424 and *UNC5B* or *SIRT4* expression levels in bladder cancer tissues further supported the negative regulation of *UNC5B* and *SIRT4* by miR-424 in vivo. Additionally, siRNA-mediated silencing of either *UNC5B* or *SIRT4* resulted in a significant decrease in the sensitivity to CDDP of bladder cancer 5637 and T24 cells, whereas miR-424-mediated decrease in CDDP sensitivity was partially restored by forced expression of UNC5B or SIRT4. Thus, these results indicate that *UNC5B* and *SIRT4* play a vital role in the regulation of CDDP-mediated apoptotic response in bladder cancer cells. In support of this notion, it has been shown that UNC5B has an ability to potentiate tumor suppressor p53-dependent apoptotic cell death in response to DNA damage [17]. UNC5B, a dependence receptor, was capable to trigger apoptotic response in the absence of its ligand netrin-1 by the activation of DAPK (death associated protein kinase) through PP2A (protein phosphatase 2A)-mediated its dephosphorylation, which subsequently induced caspase-3 activation [18]. Meanwhile, several lines of evidence indicate that SIRT4, which is localized in mitochondria, acts as a tumor suppressor [19–23]. From their results, SIRT4 participated in DNA damage-mediated metabolic responses and repressed mitochondrial glutamine metabolism [19]. Notably, it has been shown that *SIRT4*-knockout mice spontaneously develop several types of tumors and SIRT4 expression is significantly down-regulated in certain human cancers [19]. Together with these findings, our results suggest that miR-424-mediated decrease in the expressions of tumor suppressor *UNC5B* and *SIRT4* play a pivotal role in the acquisition and/or the maintenance of CDDP resistance in bladder cancer.

In accordance with the previous observations [9–12], our present observations demonstrated that HIF-1α is required for CDDP-mediated transactivation of miR-424 in bladder cancer cells. CDDP treatment in bladder cancer 5637 cells induced miR-424 expression in a HIF-1α-dependent manner under normoxia, which was consistent with the recent study describing that miR-424 is implicated in the resistance to doxorubicin and etoposide of colon cancer HCT116 cells and malignant melanoma A375 cells under normoxia [15]. It has been also shown that certain anti-cancer drugs contribute to the acquisition of the chemo-resistance through up-regulation of HIF-1α under normoxia [9–12]. Indeed, our present results revealed that CDDP treatment increases HIF-1α protein level, its recruitment onto the putative HIF-1α-binding site of miR-424 and enhances its transcriptional activity in bladder cancer cells under normoxia. Therefore, it is suggestive that HIF-1α/miR-424/UNC5B/SIRT4 regulatory axis contributes to the acquisition and/or the maintenance of CDDP resistance of bladder cancer cells irrespective of hypoxia. In other words, current study suggests that combination chemotherapy with HIF-1a inhibition would target the normoxic bladder cancer cells, which may help to optimize conventional chemotherapy with HIF-1 inhibitors. However, due to the important function of HIF signaling pathway in stimulating red blood cell proliferation, a common side effect of HIF inhibitors is anemia, which will also affect their safety in the treatment of cancer patients. Therefore, it is necessary to carry out further clinical exploration and safety assessment.

## Conclusions

In summary, our current results indicate that CDDP-induced expression of miR-424 decreases the sensitivity to CDDP of bladder cancer cells through down-regulation of pro-apoptotic *UNC5B *and *SIRT4*, and thus miR-424 is a promising molecular target for the effective treatment of the bladder cancer patients. From the clinical point of view, it is highly likely that the combination chemotherapy of CDDP plus HIF-1α/miR-424 inhibition might have a significant impact on hypoxic as well as normoxic bladder cancer cells.

## Supplementary information

**Additional file 1: Supplementary Table 1.** Sequences of oligonucleotide primers used for qPCR in the study. **Supplementary Table 2.** Clinicopathological characters of the patients with bladder cancer in our dataset.

## Data Availability

The datasets used and/or analysed during the current study are available from the corresponding author on reasonable request.

## Abbreviations

ChIP: Chromatin immunoprecipitation
*SIRT4*: Sirtuin4
CDDP: Cisplatin
DOX: Doxorubicin
VP16: Etoposide
L-PAM: Melphalan
5-FU: 5-flouoruracil
GEM: Gemcitabine
DTX: Docetaxel
HIF-1α: Hypoxia-inducible factor-1α
HREs: Hypoxia-responsive elements
miRNAs: MicroRNAs
FBS: Fetal bovine serum
qRT-PCR: Quantitative real-time PCR
TCGA: The Cancer Genome Atlas
WHO: World Health Organization
PTX: Paclitaxel
CBDCA: Carboplatin
