# Supplementary material for: HIF-1α-dependent miR-424 induction confers cisplatin resistance on bladder cancer cells through down-regulation of pro-apoptotic UNC5B and SIRT4
Source: J Exp Clin Cancer Res. 2020 Jun 10;39:108. doi: 10.1186/s13046-020-01613-y (PMC7285474; doi:10.1186/s13046-020-01613-y)
Supplement: Supplementary file 1 — Additional file 1: Supplementary Table 1. Sequences of oligonucleotide primers used for qPCR in the study. Supplementary Table 2. Clinicopathological characters of the patients with bladder cancer in our dataset. [file 13046_2020_1613_MOESM1_ESM.doc]

Supplementary Table 1. Sequences of oligonucleotide primers used for qPCR in the study

| **Gene name** | **Oligonucleotide sequence (5′ to 3′)** |
| --- | --- |
| UNC5B | Fwd GGGCTGGAGGATTACTGGTG |
| UNC5B | Rev TGCAGGAGAACCTCATGGTC |
| SIRT4 | Fwd ATGTGGATGCTTTGCACACCAAGG |
| SIRT4 | Rev TTCAGGACTTGGAAACGCTCTTGC |
| RPL13A | Fwd CTCAAGGTCGTGCGTCTG |
| RPL13A | Rev TGGCTTTCTCTTTCCTCTTCT |
| CA9 | Fwd TCTCGTTTCCAATGCACGTACAGC |
| CA9 | Rev AGTGACAGCAGCAGTTGCACAGT |
| GAPDH | Fwd CTGCACCAACAATATCCC |
| GAPDH | Rev GTAGAGACAGGGTTTCAC |
| MiR-424 | Fwd GCGGCGGCAGCAGCAATTCATG |
| MiR-424 | Rev ATCCAGTGCAGGGTCCGAGG |
| pri-MiR-424 | Fwd GCTTCCTTCAGTCATCCAGTCTT |
| pri-MiR-424 | Rev AAACATGAATTGCTGCTGTATCC |
| U6 | Fwd GGAACGATACAGAGAAGATTAGCA |
| U6 | Rev GTGCAGGGTCCGAGGT |

Supplementary Table 2. Clinicopathological characters

**of the patients with bladder cancer in our dataset**

|  | **Group** | **Cases** |
| --- | --- | --- |
| Gender | Male | 22 |
|  | Female | 8 |
|  |  |  |
| Age (years) | ≥60 | 23 |
|  | <60 | 7 |
|  |  |  |
| Pathologic T status | Ta-T1 | 6 |
|  | T2-T4 | 24 |
|  |  |  |
| Grade | High | 22 |
|  | Low | 8 |
|  |  |  |
| Metastasis | Yes | 5 |
|  | No | 25 |
